# Development and validation of an indirect competitive lateral flow immunoassay for the detection of acetaminophen (paracetamol) in bovine urine

**DOI:** 10.1007/s00216-024-05721-y

**Published:** 2025-01-07

**Authors:** Samantha Sasse, Ariadni Geballa-Koukoula, Toine F. H. Bovee

**Affiliations:** Wageningen Food Safety Research (WFSR), Part of Wageningen University & Research, Wageningen, The Netherlands

**Keywords:** Paracetamol, Residues, Animal-derived matrices, Indirect competitive lateral flow immunoassay, Carbon nanoparticles, On-site monitoring

## Abstract

Paracetamol (PCM) is a commonly used analgesic and antipyretic agent for humans worldwide. However, PCM overdoses or overuse can cause health issues, such as hepatoxicity. As PCM is also used for the treatment of farm animals, it is essential to monitor these residues in animal-derived matrices at risk-based sites in order to minimize the intake of PCM through the food chain. In the present study, we have developed a novel carbon nanoparticle (CNP)-based indirect competitive lateral flow immunoassay (icLFIA) for the rapid detection of PCM in bovine urine. The developed icLFIA can detect PCM residues within 10 min, and its performance was validated according to Commission Implementing Regulation (EU) 2021/808, i.e., determination of the detection capability (CCβ), specificity, robustness, and stability. The CCβ of the icLFIA for PCM in bovine urine is 5 mg/L and the icLFIA is proven to be selective and specific towards PCM in bovine urine, as no matrix interference and cross-reactivity were observed, except for high concentrations of orthocetamol. The icLFIA for PCM in bovine urine is robust to (small) variations in reading time, but it remains necessary to strictly use a dilution ratio of running buffer/bovine urine of 80/20. Moreover, the produced icLFIAs are stable for at least 56 days under the stored conditions. In conclusion, the developed and validated icLFIA provides a rapid and cost-effective method for on-site monitoring of PCM abuse in cattle.

**Supplementary Information:**

The online version contains supplementary material available at 10.1007/s00216-024-05721-y.

## Introduction

Since its introduction in 1950, paracetamol (PCM) has become one of the most popular and inexpensive over-the-counter analgesic and antipyretic agents for humans worldwide. PCM is a first-line medicine for the treatment of fever, flu, and colds, as well as mild-to-moderate pain conditions, such as headache, sore throat, earache, muscle pain, menstrual pain, and osteoarthritis. Although PCM is generally considered safe compared to non-steroidal anti-inflammatory drugs (NSAIDs) for the treatment of similar conditions, exceeding recommended therapeutic doses can still lead to adverse health effects in humans, such as hepatoxicity or even death [1–3].

Apart from direct intake of PCM through medication, the population may also be exposed to PCM through residues in the food chain or through environmental sources of aniline or related compounds that are metabolized into PCM. In fact, PCM has been found in the urine of PCM users as well as individuals of known occupational or unknown source exposure to aniline [4]. Additionally, PCM was detected in urine from untreated veal calves, which could be explained by low levels of PCM found in dairy-based calf milk replacer and roughage [5]. For farm animals, PCM is only permitted for oral use in pigs, but no maximum residue limit (MRL) has been set and no withdrawal period between the last administration of a veterinary medicine and the slaughter or production of food from an animal applies [6]. However, it is suspected that PCM is also used in cattle to reduce the symptoms of pain and illnesses before they are transported to the slaughterhouse. In this way, the unsuitability of these farm animals for transport and entry checks at the slaughterhouse could be masked.

To minimize the public health risks related to misuse of PCM, it is important to monitor PCM residues in the food chain. Typically, PCM residues in animal-derived matrices, such as muscle, liver, kidney, and urine, are determined by liquid chromatography coupled to tandem mass spectrometry (LC-MS/MS) [7–9]. Some of these studies have also included the main conjugated metabolites of PCM known as PCM sulfate and PCM glucuronide, and the isomers of PCM known as metacetamol and orthocetamol. Although LC-MS/MS provides sensitive, accurate, and confirmatory results, it is not suited for on-site analysis and early detection of food safety risks. Therefore, it is essential to use a strategy that combines the simplicity, rapidness, on-site and high-throughput analysis of screenings assays or sensors with the advantages of confirmation methods in order to move towards more risk-based monitoring and enforcement. For on-site monitoring of PCM in the food chain, urine is the most accessible animal-derived matrix that provides accurate results. The main practical benefit of directly using an assay or sensor for on-site qualitative screening is the negative outcomes, leading to reduction of sample transport to the laboratory and reduction of confirmatory analyses. Most samples will elicit negative outcomes, meaning that the farmer did not administer PCM to the animals and that the products, e.g., muscle, of these animals can be consumed safely. Only the suspect samples will be sent to a laboratory for analytical confirmation (e.g., by LC-MS/MS analysis), and if confirmed in the laboratory, the farmer may be fined and/or the products of the animal are taken from the market.

In the last decades, various screening assays and sensors have been developed for the detection of PCM, such as the enzyme-linked immunosorbent assay (ELISA) [10, 11], lateral flow immunoassay (LFIA) [12], fluorescence polarization immunoassay (FPIA) [13], immunochromatographic assay (ICA) [14], electrochemical sensor [15, 16], and surface-enhanced Raman spectroscopy (SERS) [17, 18]. Various signal indicators have been utilized in these detection methods to enhance the sensitive detection of PCM, among others, horseradish peroxidase in ELISA [11], gold nanoparticles in SERS [18], palladium nanoparticles in electrochemical sensor [16], and fluorescein-labeled analogs in FPIA [13]. However, neither of the screening assays and sensors has been developed for PCM in animal urine, nor utilize “simple” carbon nanoparticles (CNPs) for sensitive visual detection on a LFIA. CNPs were firstly introduced by Posthuma-Trumpie *et al.* [19] as a versatile signaling indicator for rapid diagnostic assays in 2012 and have been used for applications, such as the detection of species adulteration in milk [20] and biosensing chlorpyrifos in environmental water samples [21] by a LFIA.

In the present study, a novel indirect competitive lateral flow immunoassay (icLFIA) was developed for the detection of PCM in bovine urine, employing goat anti-mouse IgG antibodies coupled to carbon nanoparticles (GAM-CNP) as the signal indicator. The readout of the results was performed visually by the naked eye and by a handheld optical detector, i.e., the Cube Reader. For point-of-need applicability, the icLFIA was further integrated into a lateral flow device (LFD). Finally, to assess the performance of the point-of-need test, the LFD was validated as a qualitative screening method at a relevant level of 5 mg/L according to Commission Implementing Regulation (EU) 2021/808 [22].

## Materials and methods

### Chemicals and materials

PCM, PCM sulfate, PCM glucuronide, orthocetamol, metacetamol, diclofenac, meloxicam, and tetracycline were purchased from Sigma-Aldrich (St. Louis, MO, USA). The stock solutions and dilutions were prepared in methanol from Actu-All Chemicals (Oss, the Netherlands). Mouse anti-PCM monoclonal antibody (mAb) (2 mg/mL) and PCM-bovine serum albumin (PCM-BSA) conjugate (4 mg/mL) were purchased from Ecalbio (Wuhan, China) and the goat anti-mouse IgG FcY (GAM)-specific polyclonal antibodies (1.3 mg/mL, 115–005–071) and donkey anti-goat IgG (DAG) polyclonal (H + L) antibodies (1.3 mg/mL, 705–005-003) from Jackson ImmunoResearch (Sanbio, Uden, The Netherlands). Spezial Schwartz 4 CNPs were purchased from Orion Engineered Carbons GmbH (Eschborn, Germany). Zeba™ spin desalting columns (7 K MWCO) were obtained from Thermo Scientific (Rockford, IL, USA). BSA, Tween-20, and trehalose were acquired from Sigma-Aldrich (Zwijndrecht, The Netherlands); boric acid and sodium tetraborate from VWR (Leuven, Belgium); and phosphate-buffered saline (PBS) tablets from Millipore Corporation (Cork, Ireland). The buffers were prepared in deionized water from a Milli-Q® system with a minimum resistance of at least 18.2 MΩ cm^−1^ (Millipore, Billerica, MA, USA). The 20-mm glass fiber-based pads (Grade 8950), proprietary fiber blend-based sample pad (Grade 1660), and 15-mm cotton fiber-based wicking pads (Grade 222) were obtained from Ahlstrom (Helsinki, Finland); 25-mm Unisart® CN 95 nitrocellulose membranes from Sartorius Stedim Biotech GmbH (Gottingen, Germany); and the silica absorbent pads from Sigma-Aldrich (Zwijnrecht, The Netherlands). Backing cards and aluminum foil pouch bags for storage of the produced icLFIAs were purchased from Kenosha (Amstelveen, the Netherlands) and the 96-well microtiter plates from Greiner bio-one (Alphen a/d Rijn, The Netherlands). Cassettes for the construction of the LFD were produced using in-house 3D printing facilities.

### Preparation of CNPs functionalized with GAM antibodies

CNPs were functionalized with GAM antibodies as described previously by Sharma et al*.* [20]. Briefly, a 1% (w/v) aqueous CNP suspension was sonicated for 1 h at 40 kHz at room temperature. Next, a 0.2% (w/v) CNP suspension was prepared in 5 mM borate buffer (pH 8.8) containing boric acid and sodium tetraborate, and sonicated for 5 min. Before functionalization, the purified GAM antibodies were desalted and buffer exchanged with 5 mM borate buffer (pH 8.8) using 0.5-mL Zeba™ spin columns. Subsequently, the antibody concentration was measured with a DS-11 FX spectrophotometer for microvolumes (DeNovix, Wilmington, NC, USA). Afterwards, 0.35 mg GAM antibodies (e.g., 0.35 mL of a 1 mg/mL desalted and buffer exchanged GAM solution) were added dropwise to 1 mL of 0.2% carbon suspension. The content was gently mixed overnight with a magnetic stirrer at 200 rpm and 4 °C. After incubation, washing buffer (5 mM borate buffer, pH 8.8 containing 1% w/v BSA) was added dropwise to the suspension. The suspension was then stirred for 5 min and centrifuged at 13,600 × g for 15 min at 4 °C followed by collecting the pellet and discarding the supernatant. The washing procedure was repeated three times before the pellet was resuspended in storage buffer (100 mM borate buffer, pH 8.8 containing 1% w/v BSA) to a final concentration of 0.2% (w/v) CNPs. The GAM-CNP conjugate was stored at 4 °C until further use. The performance of the coupling procedure has been demonstrated by quantitative computer image analysis and was reported previously [23].

### Method setup by implementation of spot-based icLFIAs

The initial setup of the icLFIA for PCM was performed by manually spotting the bioreagents onto the nitrocellulose membrane. For this purpose, the nitrocellulose membrane was secured on a backing card as support and slightly overlaid with a wicking pad followed by cutting the card with all parts into 4-mm strips using the BioDot CM5000 Guillotine Cutter (Irvine, CA, USA). Subsequently, 0.5 μL of PCM-BSA (test dot) and 0.5 μL of DAG antibody (control dot) were pipetted on top of the cut nitrocellulose membrane and let dry for 2 h at room temperature. PCM-BSA conjugate was diluted to 0.125, 0.25, and 0.5 mg/L in PBS buffer, whereas the DAG antibody was diluted to 0.15 mg/mL in PBS buffer. Next, a dilution series of the anti-PCM mAb (1:250–1:2000) was prepared in running buffer composed of 0.01 M PBS (pH 7.4), 0.05% v/v Tween-20, and 1% w/v BSA. Additionally, PCM standard solutions in the concentration of 0, 0.01, and 1 mg/L were prepared. 1 μL anti-PCM mAb and 1 μL GAM-CNP conjugate were added to 98 μL diluted PCM standard solutions in a microtiter plate and gently mixed. The spot-based icLFIAs were put into the microtiter plate and developed for 10 min.

### Production of line-based icLFIAs

For the line-based icLFIAs, the test line with the PCM-BSA conjugate (0.5 mg/mL) and the control line with the DAG antibody (0.15 mg/mL) were sprayed in PBS buffer onto a nitrocellulose membrane at a rate of 1 µL/cm using a BioDot XYZ3060 dispenser (Chichester, UK). The nitrocellulose membrane sheet was let dry for 2 h at room temperature.

Next, a mixture of 1000 times diluted anti-PCM mAb in running buffer (0.01 M PBS, pH 7.4 containing 0.05% v/v Tween-20 and 1% w/v BSA) was prepared. Subsequently, the anti-PCM mAb (1:1000) and the GAM-CNP conjugate were diluted 10 times in spraying buffer (0.01 M PBS, pH 7.4 containing 10% w/v trehalose and 1% w/v BSA). Glass fiber-based antibody and conjugate pads were prepared by pipetting the diluted mixtures of anti-PCM mAb and GAM-CNP on glass fiber pads, respectively. Finally, the pads were let dry overnight.

**Fig. 1:**
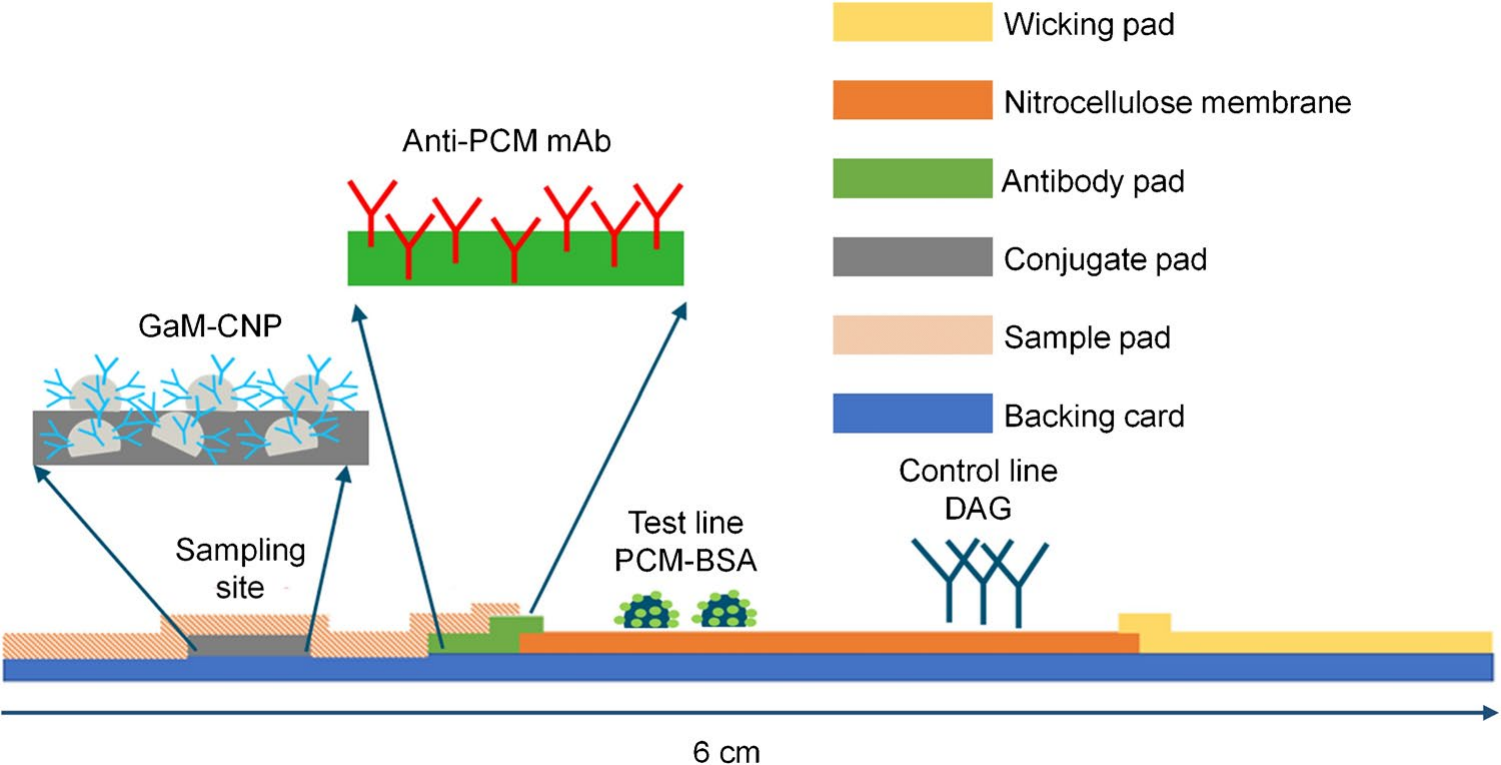
Construction of icLFIAs for PCM

For the assembly of the complete icLFIA, the dried nitrocellulose membrane with the test and control lines was secured on a backing card as support. Next, the wicking pad was placed by the end of the icLFIA, and the glass fiber-based antibody pad was placed at the beginning of the icLFIA, both slightly overlapping with the nitrocellulose membrane for approximately 1 mm. Then, the conjugate pad was secured directly on the backing support at a 2-mm distance from the antibody pad. Finally, a proprietary fiber blend-based sample pad was placed on top of the antibody and conjugate pads (Fig. 1). The backing card with all parts was cut into 4-mm strips using the BioDot CM5000 Guillotine Cutter (Irvine, CA, USA). The cut icLFIAs were stored in special bags with a silica pad and closed by a sealing apparatus until further use.

### Point-of-need LFD test for PCM in bovine urine

Before analysis, LFD was generated by placing the complete icLFIAs into a dedicated 3D printed cassette. Samples were prepared by pipetting 240 μL of running buffer and 60 μL bovine urine (ratio 80/20) in a 1.0-mL Eppendorf tube and the tube was vortexed for 5 s. Next, 100 μL of the homogeneous solution was pipetted onto the LFD and developed for 10 min.

### Validation of the point-of-need LFD test

#### Detection capability for screening (CCβ)

Endogenous PCM levels may be present in bovine urine samples. However, the maximal endogenous PCM level is not exactly known and neither is the minimal PCM level in the urine of cattle treated with PCM. Based on previous animal tests and other positive results found in bovine urines within WFSR, the screening target concentration of 5 mg/L was selected as the threshold from which PCM levels are expected in treated animals.

To determine the CCβ, 22 different batches of in-house provided samples from bovine urine, previously confirmed to be blank with an in-house developed and validated confirmatory LC–MS/MS method, were spiked with PCM at 5 mg/L and were tested divided over three consecutive days. The technical details of the LC–MS/MS method are presented in the supplementary information file. The criterion for the detection capability is that at least 21 (95%) should be characterized as non-compliant, i.e., as a suspicious sample, by the LFD for PCM. When this is the case, the screening target concentration of 5 mg/L is the CCβ of the developed LFD test (although the exact CCβ might be lower).

#### Selectivity/specificity

To assess the selectivity, the aforementioned 22 in-house provided samples from bovine urine were tested as such divided over three consecutive days to assess the false positive rate in negative samples. Preferably, the matrix of the 22 samples from bovine urine does not influence the result of the test and only leads to negative screening results that would not require additional confirmational LC–MS/MS analysis. Secondly, the specificity, i.e., the ability of the LFD to discriminate between PCM and other structurally related PCM analytes, NSAIDs, and antibiotics, was investigated. Bovine urine samples were spiked with PCM sulfate, PCM glucuronide, orthocetamol, metacetamol, diclofenac, meloxicam, and tetracycline at 5 mg/L to conclude whether the aforementioned analytes interfere with the LFD and lead to false positive results (sample classified as suspect and marked for additional confirmational LC–MS/MS analysis). Each potential interference of structurally related PCM analytes, NSAIDs, and antibiotics was performed in three different batches of bovine urine.

#### Robustness

To determine the continued performance of the LFD under different experimental conditions, minor changes were applied to the method, which are most likely to occur during routine analyses and are expected to be critical. First, the reading time was varied between 5 and 15 min, instead of 10 min. Second, the dilution ratio for running buffer and bovine urine was varied between dilution ratios of 90/10 and 70/30 instead of 80/20. All the separate conditions were performed for blank and PCM-spiked (5 mg/L) samples in three different batches of bovine urine.

#### Stability of the icLFIAs

Since the test for PCM is intended for screening purposes and on-site analysis in fresh urine samples from farms, the stability of PCM in solution, extract, and matrix is not relevant for this application. However, the stability of the icLFIA is important as it consists of antibodies and conjugates, i.e., proteins whose functionality might be affected by light, time, and temperature, and thus storage shelf life. After production, the icLFIAs were stored in closed aluminum bags with a silica desiccant at room temperature. The bovine urine was stored at − 20 °C. The icLFIAs were tested at 1, 3, 7, 14, 28, and 56 days after production to prove that the result is not altered over time. Each time point was performed in triplicate for a blank and a PCM-spiked (5 mg/L) bovine urine sample.

### Data assessment

**Fig. 2:**
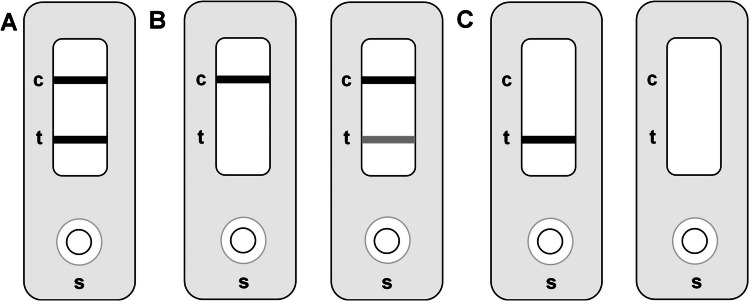
Assessment of LFDs (**A**) negative/complaint, (**B**) positive/suspect, (**C**) invalid. On the LFD cassettes, the c indicates the position the control line is expected, the t indicates the position the test line is expected and the s indicates the sampling site

The results of the icLFIAs were read by the naked eye and by a handheld optical detector, i.e., a Cube Reader (Chembio, Berlin, Germany). Images of the developed icLFIAs were recorded with a Samsung A50 smartphone in a Caruba Portable Photocube LED 70 × 70 × 70 cm (Caruba, Beilen, The Netherlands) to maintain similar lighting conditions. For the visual assessment, the appearance of both the control and the test line indicates a negative result, whereas only the appearance of the control line indicates a suspect result. The absence of the control line means that the test was not valid (Fig. 2).

The Cube Reader measures the intensity of the control and test line in arbitrary units (AU). Subsequently, the threshold (T) and the cutoff factor (Fm) could be determined based on the test line versus control line ratio as presented in Eqs. (1) and (2).

1 $$T=B-1.64 \times SDb$$

where B is the mean test/control line response of the blank samples and SDb is the standard deviation of the blank samples.

2 $$Fm=M-1.64 \times SDs$$

where M is the mean test/control line response of the spiked samples and SDs is the standard deviation of the spiked samples.

## Results and discussion

### Optimization of the icLFIA

To maximize the performance of the icLFIA, several key parameters were optimized, including the concentration of anti-PCM mAb and PCM-BSA, the type of running buffer, the type of spraying buffer for immobilization of anti-PCM mAb and GAM-CNP, the dilution of bovine urine, and the interpretation times (run or reading time).

For adequate signal intensity and sensitivity on the test line, the concentrations of anti-PCM mAb and PCM-BSA were optimized by implementing spot-based icLFIAs (see the “Method setup by implementation of spot-based icLFIAs” section). Reducing the concentration of anti-PCM mAb caused a noticeable decrease in the signal intensity of the spotted test dots (Fig. S1). The results indicated that the optimal concentrations of anti-PCM mAb and PCM-BSA, taking into account the signal intensity and sensitivity of the test dot, were 1:1000 dilution and 0.5 mg/mL, respectively.

**Fig. 3:**
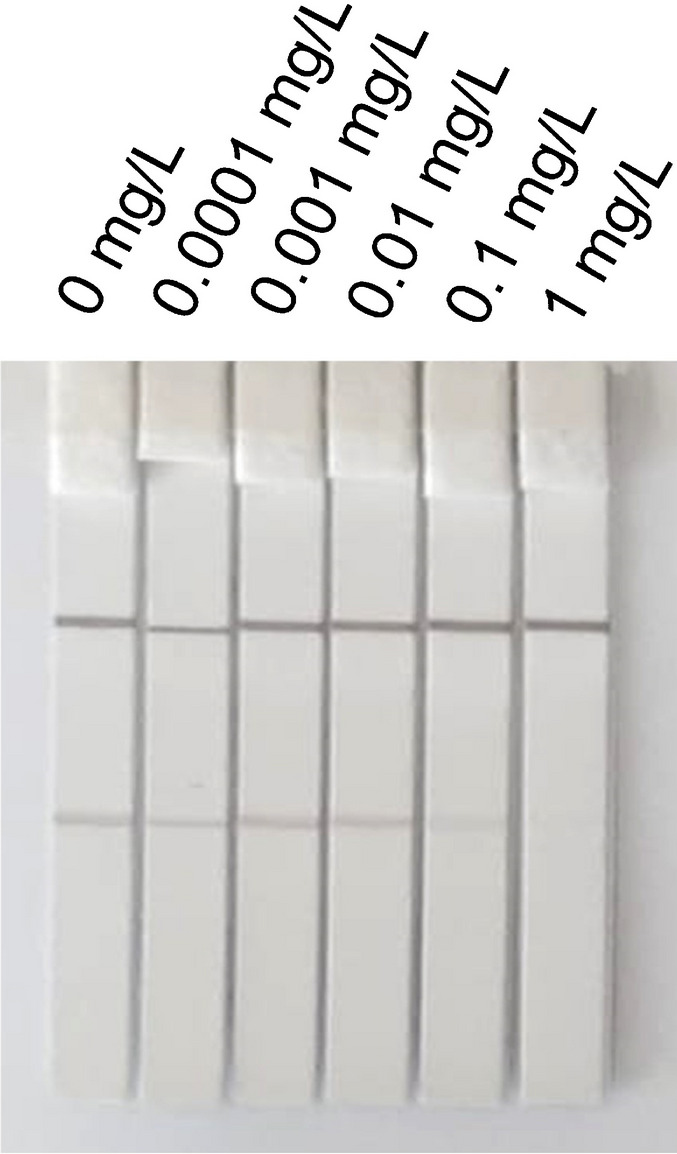
icLFIA results obtained after 10-min runs with different concentrations of PCM (0.0001–1 mg/L), using anti-PCM mAb at 0.5 mg/mL and PCM-BSA at 1:1000 dilution in 0.01 M PBS (pH 7.4), 0.05% v/v Tween-20, and 1% w/v BSA

Furthermore, the different components of the running buffer and concentrations thereof may influence the performance of the CNP-based icLFIA. For instance, buffer components and their concentrations may influence the intensity of the control and test lines, the background noise, and the degree of non-specific binding. It was observed that when borate buffer was used in the running buffer composition, it resulted in less favorable outcomes in terms of fading of the test line compared to PBS buffer (data not shown). Next, different compositions of additives in 0.01 M PBS buffer, including BSA (0.5–5%), Tween-20 (0.1–1%), and tergitol (0.1–1%), were extensively tested by applying line-based icLFIAs but with the GAM-CNP conjugate and anti-PCM mAb in solution. Several running buffer formulations were effective in reducing the test line intensity with increasing PCM concentrations, including the running buffer formulations of (i) 0.01 M PBS with 0.05% v/v Tween-20 and 1% w/v BSA, (ii) 0.01 M PBS with 0.1% v/v Tween-20 and 1% BSA, and (iii) 0.01 M PBS with 0.05% v/v Tween-20, 0.1% tergitol, and 1% BSA (Fig. S2). Ultimately, the running buffer of 0.01 M PBS (pH 7.4) with 0.05% v/v Tween-20 and 1% w/v BSA was selected as the optimum one for further experiments. A dilution series of PCM standard solution in the selected running buffer resulted in a sensitivity of approximately 0.1 to 1 mg/L when assessed by the naked eye (Fig. 3).

For the development of a LFD, the anti-PCM mAb, and the GAM-CNP were immobilized on separate glass fiber-based pads deposited at the beginning of the icLFIAs (Fig. 1). For this purpose, different components of spraying buffers and concentrations thereof were tested and developed with running buffer only to assess the release of both components from the glass fiber-based pads, as well as, the stability of the immobilized components over time. During the process or storage, these components are subjected to several stresses, including temperature changes, dehydration, or changes in pH. Herein, it is essential to include a protein stabilizer, such as trehalose or sucrose, in the spraying buffer to retain the structure, function, and activity of the components on the icLFIAs [24, 25]. The spraying buffer composed of 0.01 M PBS (pH 7.4), 10% w/v trehalose, and 1% w/v BSA was selected for further experiments as the test and control lines were most intense (Fig. S3). The influence of the dilution of bovine urine and interpretation times was investigated during the validation of the point-of-need LFD for PCM (“Robustness” section).

### Validation of the point-of-need LFD test

The validation of the LFD for PCM in bovine urine was performed according to (EU) 2021/808 [22] and with the use of the EURL Guidance Document on Screening Method Validation [26]. As the aim is to use the developed icLFIA as a qualitative screening test, the performance characteristics CCβ, selectivity/specificity, robustness, and stability were determined. The following sections present the validation results of the developed LFD for PCM in bovine urine.

#### Detection capability for screening (CCβ)

**Fig. 4:**
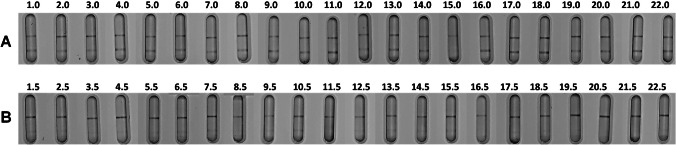
Visual LFD results of the 22 samples (**A**) 0 mg/L (blank urine) and (**B**) 5 mg/L PCM spiked urine developed for 10 min

For the determination of CCβ, the icLFIAs were produced and placed in a dedicated cassette (“Production of line-based icLFIAs” section). The urine samples (blank or 5 mg/L PCM spiked) were diluted five times in running buffer and the LFDs were then developed for 10 min (“Point-of-need LFD test for PCM in bovine urine” section). The visual results from the validation experiments of these blank and spiked bovine urine samples are shown in Fig. 4. All tests were valid, as control lines appeared on all of the 44 LFDs. All blanks, except 15.0, showed a clear test line, meaning that all samples, except 15.0, were correctly classified as negative. Finally, all the spiked samples caused a fading of the test line, indicating that all spiked samples were correctly classified as suspect.

**Fig. 5:**
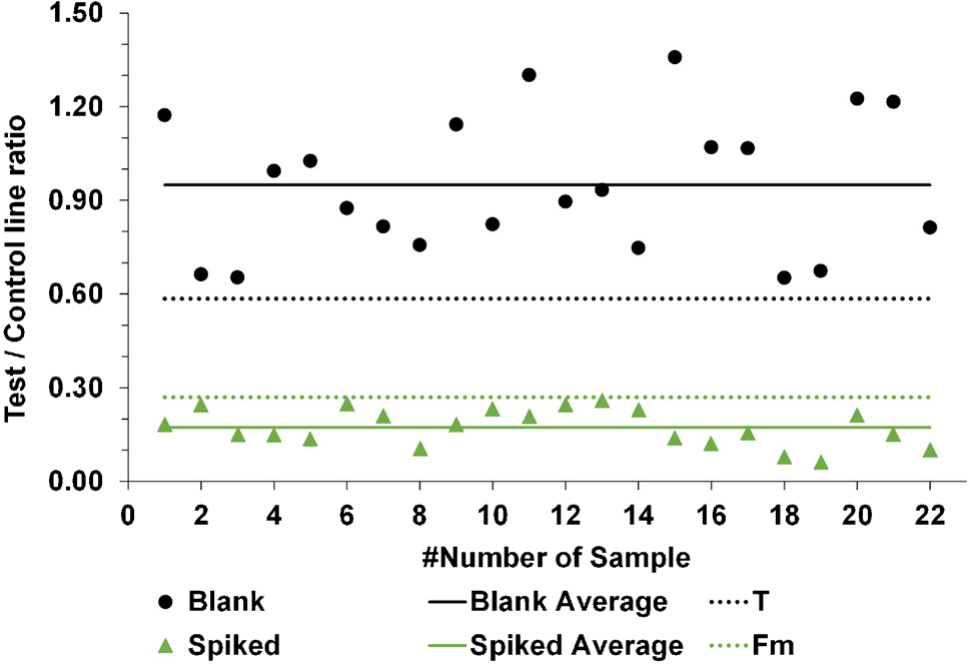
Cube readouts of the blank and PCM spiked (5 mg/L) bovine urine samples

After visual assessment, the intensity of the control and test lines was also measured with the Cube Reader. Subsequently, the Fm and T were calculated at 0.27 and 0.59, respectively (Fig. 5).

The validation is successful if Fm < T, and if less than 5% of the total number of spiked samples from bovine urine is showing a response above the determined T value. According to the Cube readouts and the calculated Fm and T values (Fig. 5), both requirements are met. All the spiked bovine urines show a response lower than T and are therefore classified as suspect. In addition, all blanks show a response higher than T and are therefore classified as negative. In conclusion, the CCβ of PCM in bovine urine is 5 mg/L for the developed icLFIA.

#### Selectivity/specificity

All the 22 blank bovine urines show a response higher than T in Fig. 5 and therefore are classified as negative. In other words, no false positive results were obtained from the LFD tests when measured with the Cube Reader and thus the method is indeed selective, i.e., no false positives due to matrix effects or other compounds present in bovine urine.

**Fig. 6:**
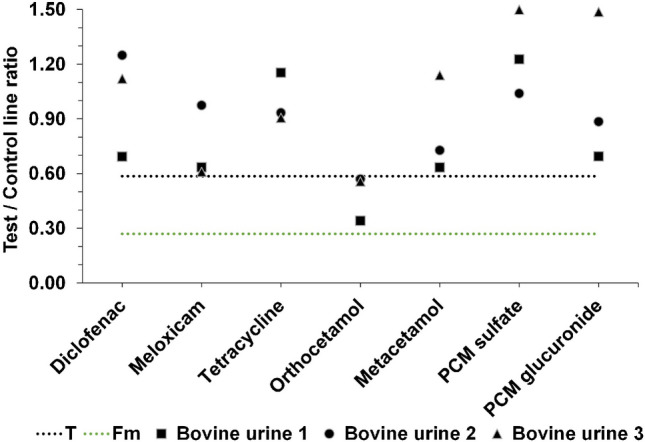
Cube readouts of the cross-reactivity tests for investigation of specificity

In addition to the examination of the blank samples, structural analogs of PCM, NSAIDs, and antibiotics in bovine urine were analyzed to further assess the specificity of the developed LFD for PCM. Hereby, three blank samples from the determination of CCβ were used to determine if these other analytes interfere with the developed LFD for PCM. From the visual evaluation of the LFDs (Fig. S4) and the processing of the Cube readouts (Fig. 6), it can be observed that a high concentration (5 mg/L) of spiked tetracycline, diclofenac, and meloxicam did not yield a suspect (false positive) result of the LFD for PCM in bovine urine. Regarding the structural analogs of PCM, no false positive results were obtained for metacetamol, PCM sulfate, and PCM glucuronide. In contrast, orthocetamol showed false positive results according to the Cube readout and the previously calculated T value (Fig. 6); however, the results could be interpreted as negative by visual assessment as seen by the observation of two lines (Fig. S4).

Overall, neither tetracycline nor NSAIDs and structural analogs of PCM cross-reacted when assessed visually, meaning that the developed icLFIA is very specific for PCM. When applying the Cube, all blanks were classified as negative/compliant and all samples containing PCM at 5 mg/L were classified as suspect. Only blanks spiked with orthocetamol resulted in suspect LFD readouts. Although the LFD is specific for PCM, there have been cases at WFSR in which higher levels of the metabolites, i.e., PCM glucuronide and PCM sulfate, have been detected in urine of treated farm animals compared to PCM. Therefore, cross-reactivity with the metabolites of PCM could have provided even a broader relevant scope of the method, although the detection of metabolites will also be covered when a suspect result arises from the LFD test and confirmation analysis with LC–MS/MS is required.

#### Robustness

**Fig. 7:**
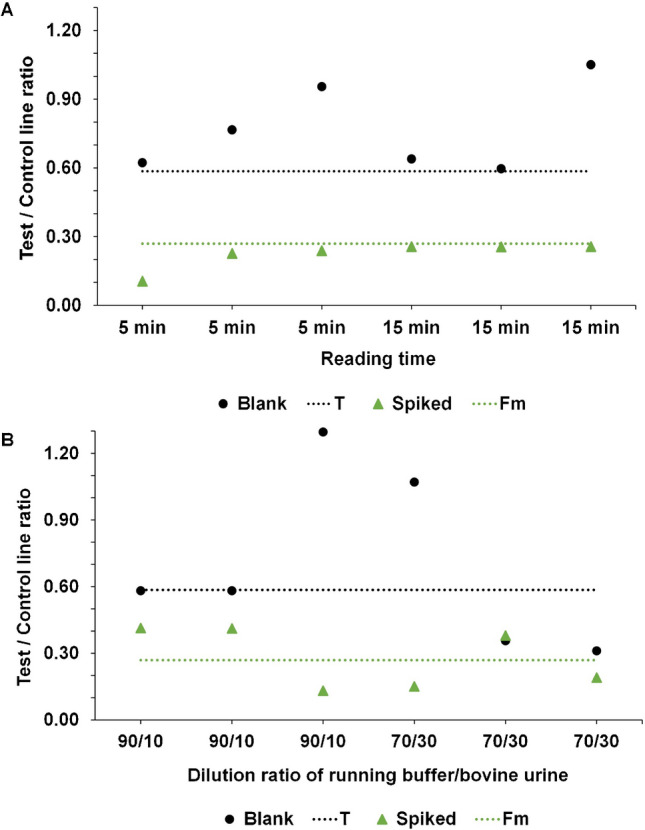
The effect of the (**A**) reading time and (**B**) dilution ratio on the Cube readout result of the LFD

To examine the robustness of the method, the most critical parameters of the experimental procedure were varied, i.e., parameters that could potentially influence the outcome of the LFD. The dilution ratio of the running buffer/bovine urine and the reading time of the LFD test were selected as the most critical parameters in routine application. The visual results of the robustness experiments (Fig. S5), as well as the related Cube readouts (Fig. 7A), show that a small difference in reading time does not influence the results. The standard reading time is 10 min; however, assessing the results after 5 or 15 min also leads to the correct classification of the samples from bovine urine.

Contrary, a minor change in the dilution ratio of the urine sample emerges to influence the result both when assessed visually (Fig. S5) and by the Cube Reader (Fig. 7B). With the Cube Reader, two out of the three blank samples were classified as suspects when using a dilution ratio of 70/30, i.e., two false positives when less diluted urine was used compared to the standard and subscribed ratio of 80/20. Thus, it is necessary to maintain the correct dilution ratio of 80/20 of running buffer/bovine urine for the execution of the LFD for PCM in bovine urine, while the reading time of 10 min is less strict, as 5- and 15-min readouts lead to the same classification of the urine samples tested. Still, the standard assay time was kept at 10 min rather than using a faster 5-min assay to ensure consistent control lines’ appearance when larger batches of urine samples are tested.

#### Stability of the icLFIAs

**Fig. 8:**
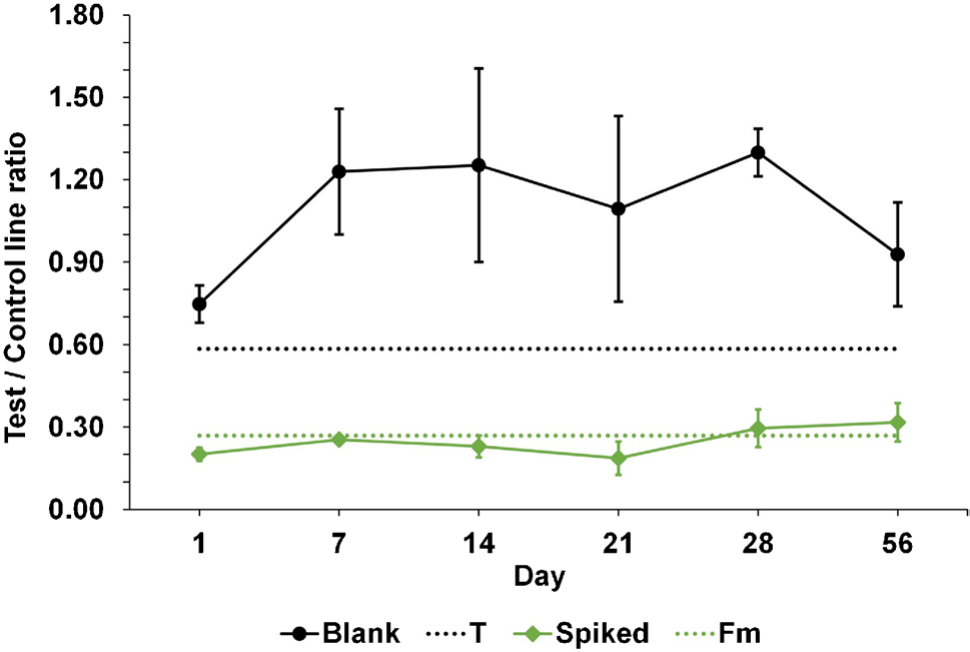
Cube readouts from the stability experiments of the icLFIA for PCM using blank and spiked (5 mg/L PCM) bovine urine samples

The stability of icLFIAs for a longer period is important, especially when reducing time and costs by preparing directly a large batch of icLFIAs and aiming to use that batch to measure PCM abuse in bovine urine over a time ranging from days to months. Figure S6 and Fig. 8 present the visual results and the Cube readouts of the stability experiments of the LFD for PCM up to 56 days using a blank and spiked (5 mg/L PCM) bovine urine sample. During this period, the icLFIAs were stored in closed aluminum bags with a silica desiccant at room temperature.

Based on these results, it can be concluded that the icLFIAs for PCM are stable for at least 56 days under the stored conditions. At day 56, the icLFIAs result in clear control and test lines for blank samples of bovine urine and the test lines still disappear when the bovine urine samples contain 5 mg/L PCM. Based on the Cube readout, the test line versus control line ratio of the blank samples from bovine urine is higher than T, i.e., negative/compliant result, and the spiked samples result in a response intensity lower than T, i.e., positive/suspect result, for all days and up to 56 days.

## Conclusions

A new icLFIA for the rapid detection of PCM in bovine urine was successfully developed and validated according to (EU) 2021/808. The requirements for CCβ were met, indicating that possible PCM abuse can be detected on-site from a relevant level (> 5 mg/L). Furthermore, the method is selective and specific, showing no matrix interference and cross-reactivity with tetracycline, NSAIDs, and metabolites and isomers of PCM, except for high concentrations of orthocetamol when based on the results from the Cube Reader. Additionally, the method is robust to small changes in reading time; however, it is necessary to strictly use a dilution ratio of running buffer/bovine urine at 80/20, i.e., five times diluted bovine urine samples. Moreover, the produced icLFIAs for the detection of PCM in bovine urine are stable for at least 56 days when stored in closed aluminum bags with a silica desiccant at room temperature. After development, the LFDs were mostly classified similarly by the naked eye and the Cube Reader. Nevertheless, the Cube Reader is strongly preferred in the assessment of the results since the human variable and bias when the result is read visually are eliminated. Furthermore, the Cube Reader is programmable with the possibility to demonstrate a message indicating “suspect or non-suspect” when certain values are read. Also, the data is recorded digitally, which is ideal for on-site purposes and further storage that could ease future trace back of samples. In conclusion, the icLFIA shows excellent potential for the timely regulation and monitoring of PCM abuse in cattle.

## Supplementary Information

Below is the link to the electronic supplementary material.

Supplementary file1 (PDF 812 KB)

## Data Availability

Data will be made available on request.
