# Supplementary material for: Development and validation of an indirect competitive lateral flow immunoassay for the detection of acetaminophen (paracetamol) in bovine urine
Source: Anal Bioanal Chem. 2025 Jan 7;417(6):1177–86. doi: 10.1007/s00216-024-05721-y (PMC11802664; doi:10.1007/s00216-024-05721-y)
Supplement: Supplementary file 1 — Supplementary file1 (PDF 812 KB) [file 216_2024_5721_MOESM1_ESM.pdf]

Supplementary information

Development and validation of an indirect competitive lateral flow immunoassay for the detection of acetaminophen (paracetamol) in bovine urine

Samantha Sasse[1*], Ariadni Geballa-Koukoula[1*], Toine Bovee[1]

[1]Wageningen Food Safety Research (WFSR), Part of Wageningen University & Research, Wageningen, The Netherlands

Corresponding author: Samantha Sasse
E-mail: samantha.sasse@wur.nl

*S.S. and A.G-K. contributed equally

Supplementary details of the confirmatory LC-MS/MS method

LC conditions

Water Acquity pump and injection system.

Analytical column: Acquity UPLC CSH C18 (100 mm x 2.1 mm, 1.7 µm).

Mobile phase A: 0.1% formic acid in Milli-Q water.
Mobile phase B: 0.1% formic acid in acetonitrile.

Table S1 Gradiënt program

Time (min)	Mobile phase A (%)	Mobile phase B (%)
0.0	100	0
1.0	100	0
2.5	90	10
5.0	80	20
5.5	0	100
6.5	0	100
6.6	100	0
7.0	100	0

Flow: 0.4 mL/min
Column temperature: 40 °C
Injection volume: 10 µL

MS conditions

Sciex Q6500 with electrospray (ESI) interface.

Table S2 MS/MS fragmentation conditions in ESI positive mode

Analyte	Precursor ion *m/z*	Product ion *m/z*	DP (V)	CE (V)	CXP (V)
Paracetamol	151.9	93.2	35	40	10
		110.2	35	15	10
		65.3	35	55	10
Paracetamol-d3	154.9	93.2	35	40	10
Paracetamol glucuronide	327.9	110.2	60	40	10
		152.1	60	15	10
Paracetamol glucuronide-d3	330.9	113.2	60	15	10
		155.1	60	15	10
Paracetamol sulphate	229.8	107.0	-35	-40	-10
		150.0	-35	-22	-10
Paracetamol sulphate-d3	232.8	107.0	-35	-40	-10
		153.0	-35	-22	-10

Supplementary figures

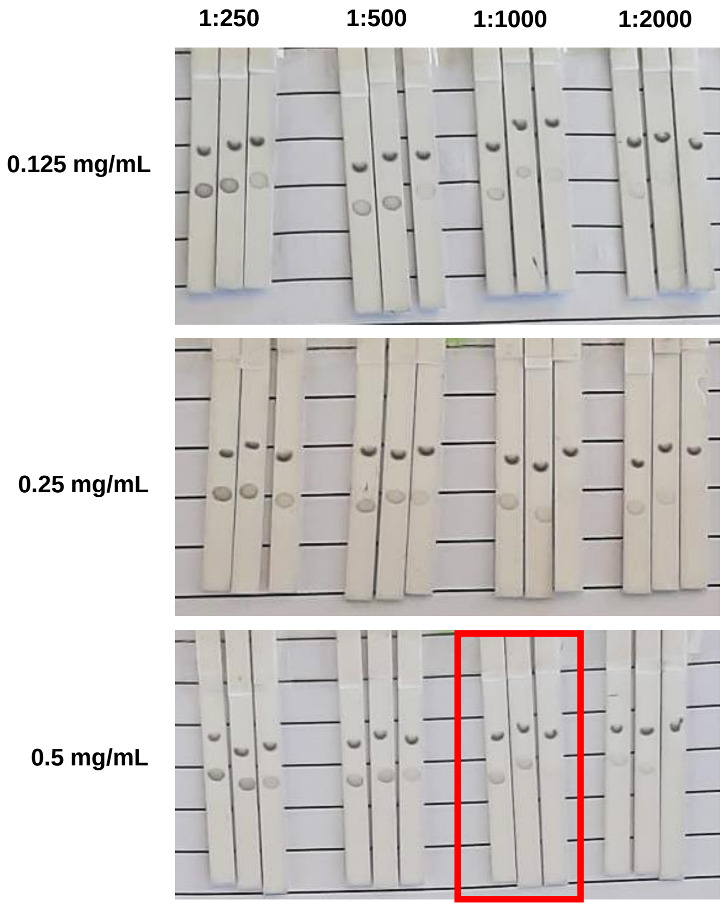

Fig. S1 Different PCM-BSA concentrations (vertical) and anti-PCM mAb dilutions (horizontal) after development of LFIAs for 10 minutes. LFIAs were developed from left to right with blank, 10 ppb and 1 ppm PCM concentrations for each combination of PCM-BSA and anti-PCM mAb. Red circled was selected for further experiments

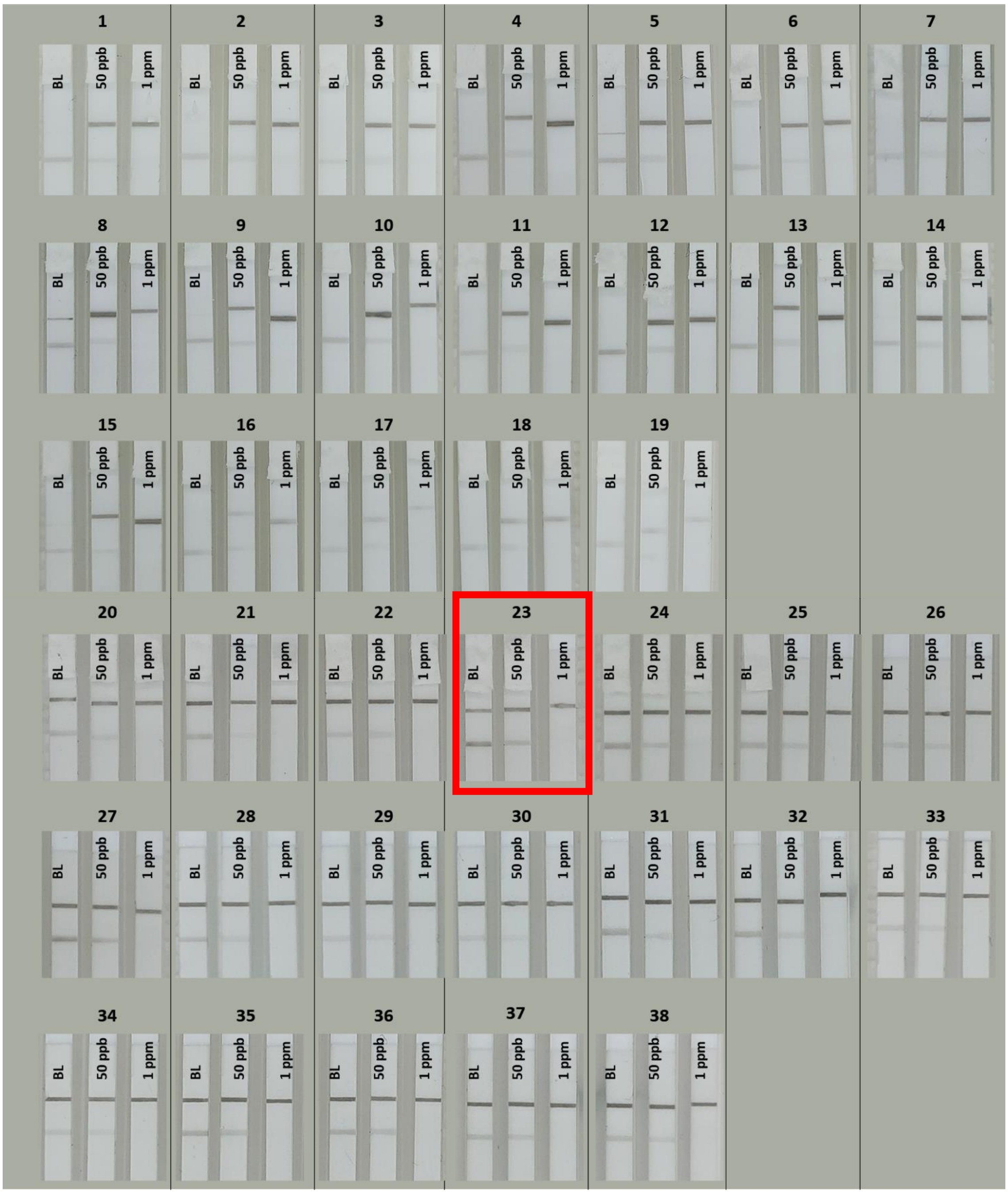

Fig. S2 Optimalization of running buffer consisting of (1-38) 0.01 M PBS with (1-19) 0.5% BSA or (20-38) 1% BSA and (1, 20) 0.1% tergitol, (2, 21) 0.5% tergitol, (3, 22) 1% tergitol, (4, 23) 0.05% Tween-20, (5, 24) 0.05% Tween-20 + 0.1% tergitol, (6, 25) 0.05% Tween-20 + 0.5% tergitol, (7, 26) 0.05% Tween-20 + 1% tergitol, (8, 27) 0.1% Tween-20, (9, 28) 0.1% Tween-20 + 0.1% tergitol, (10, 29) 0.1% Tween-20 + 0.5% tergitol, (11, 30) 0.1% Tween-20 + 1% tergitol, (12, 31) 0.5% Tween-20, (13, 32) 0.5% Tween-20 + 0.1% tergitol, (14, 33) 0.5% Tween-20 + 0.5% tergitol, (15, 34) 0.5% Tween-20 + 1% tergitol, (16, 35) 1% Tween-20, (17, 36) 1% Tween-20 + 0.1% tergitol, (18, 37) 1% Tween-20 + 0.5% tergitol or (19, 38) 1% Tween-20 + 1% tergitol. Red circled was selected for further experiments

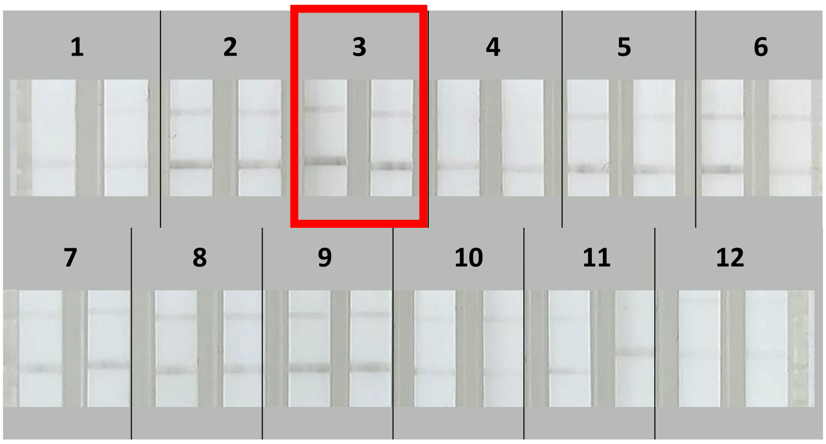

Fig. S3 Optimalization of spraying buffer consisting of (1-12) 0.01 M PBS with (1) 1% BSA + 1% trehalose, (2) 1% BSA + 5% trehalose, (3) 1% BSA + 10% trehalose, (4) 2% BSA + 1% trehalose, (5) 2% BSA + 5% trehalose, (6) 2% BSA + 10% trehalose, (7) 1% BSA + 1% sucrose, (8) 1% BSA + 5% sucrose, (9) 1% BSA + 10% sucrose, (10) 2% BSA + 1% sucrose, (11) 2% BSA + 5% sucrose or (12) 2% BSA + 10% sucrose. Red circled was selected for further experiments

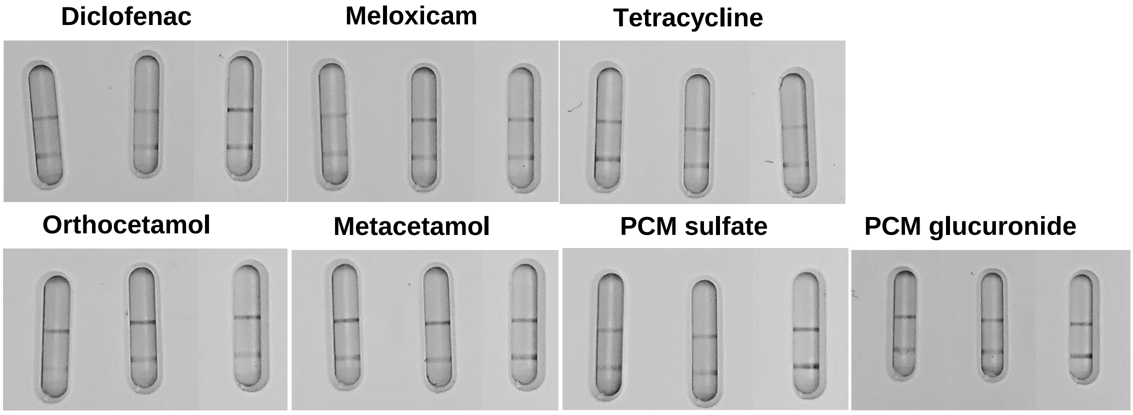

Fig. S4 Visual results from specificity tests, i.e. cross-reactivity with diclofenac, meloxicam, tetracycline, orthocetamol, metacetamol, PCM sulfate, PCM glucuronide at 5 mg/L

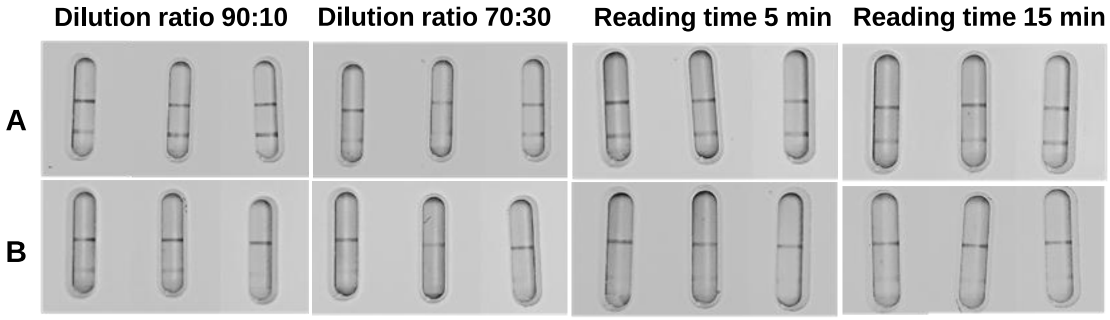

Fig. S5 Visual results from robustness tests of three different batches of bovine urine with (A) 0 and (B) 5 mg/L spiked PCM

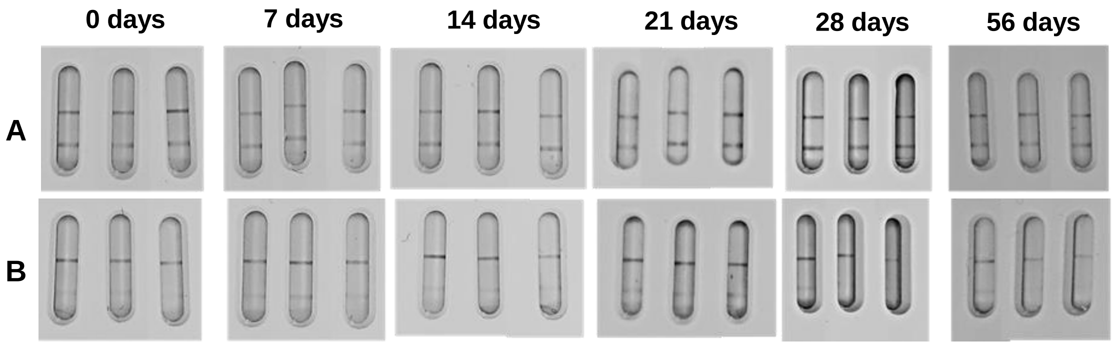

Fig. S6 Visual results from the stability tests of three different batches of bovine urine with (A) 0 and (B) 5 mg/L spiked PCM
